# Is there a case for cisplatin in the treatment of small-cell lung cancer? A meta-analysis of randomized trials of a cisplatin-containing regimen versus a regimen without this alkylating agent

**DOI:** 10.1054/bjoc.2000.1164

**Published:** 2000-06-02

**Authors:** J-L Pujol, Carestia, J-P Daurès

**Affiliations:** Départment de Biostatistiques Epidemiologie et Recherche Clinique, Institut Universitaire de Recherche Clinique, Rue de la Cardonille, Montpellier Cedex 5, 34093, France; Départment d'Oncologie Thoracique, Centre Hospitalier Universitiare de Montpellier, Hôpital Arnaud de Villeneuve, Montpellier Cedex, 34295, France

**Keywords:** SCLC, chemotherapy, cisplatin, meta-analysis

## Abstract

Chemotherapy is the backbone of small-cell lung cancer therapy. However, optimal drug combinations and schedules remain to be defined and there is hitherto no world-wide accepted standard regimen. Cisplatin, an alkylating agent with high putative toxicity is currently widely used although its effectiveness in this disease has not been established firmly. We conducted a meta-analysis of published data reporting trials randomizing a cisplatin-containing regimen versus a regimen without this alkylating agent in order to determine possible differences in survival response and toxicity. Nineteen trials have been identified in medical literature (4054 evaluable patients). Ten trials randomized patients to receive a cisplatin-etoposide regimen versus a regimen without any of these two drugs. A subgroup analysis was, therefore, carried out in the nine remaining trials that randomly allocated patients between two regimens differing in the absence or presence of cisplatin, whereas etoposide was given (or not given) in both arms (1579 evaluable patients). The DerSimonian and Laird method was used to estimate the size effects and the Peto and Yusuf method was used in order to generate the odds ratios (OR) of reduction in risk of death and the increase in probability of being responders to chemotherapy. There was no significant difference between the cisplatin-containing regimen and the regimen without this drug when the risk of toxic-death was taken into account with respective probabilities of 3.1 and 2.7% (NS). Patients randomized in a cisplatin-containing regimen had an increase in probability of being responders with an OR of 1.35, 95% confidence interval (CI) of 1.18–1.55;*P*< 10^–5^corresponding to an increase of objective (partial plus complete) response rate from 0.62 to 0.69 (a result taking into account a significant heterogeneity). Patients treated with a cisplatin-containing regimen benefited from a significant reduction of risk of death at 6 months and 1 year with respective OR 0.87, 95% CI 0.75–0.98, *P* = 0.03, and or 0.80, 95% CI 0.69–0.93, *P* = 0.002 (no statistical heterogeneity). This corresponded to a significant increase in the probability of survival of 2.6% and 4.4% at 6 months and 1 year respectively. The meta-analysis restricted to the subset of nine trials without etoposide treatment imbalance reached similar conclusions. A cisplatin-containing regimen yields a higher response rate and probability of survival than does a chemotherapy containing others alkylating agents without a perceptible increase in risk of toxic-death. © 2000 Cancer Research Campaign


*British Journal of Cancer* (2000) **83**(1), 8–15
© 2000 Cancer Research Campaign
doi: 10.1054/ bjoc.2000.1164, available online at http://www.idealibrary.com on IDEAL®

### Is there a case for cisplatin in the treatment of small-cell lung cancer? A meta-analysis of randomized trials of a cisplatin-containing regimen versus a regimen without this alkylating agent

**J-L Pujol[1,2], Carestia[1,2*] and J-P Daurès[1]**

[1]Départment de Biostatistiques Epidemiologie et Recherche Clinique, Institut Universitaire de Recherche Clinique, Rue de la Cardonille, 34093 Montpellier Cedex 5, France; [2]Départment d'Oncologie Thoracique, Centre Hospitalier Universitiare de Montpellier, Hôpital Arnaud de Villeneuve, 34295 Montpellier Cedex, France

**Summary** Chemotherapy is the backbone of small-cell lung cancer therapy. However, optimal drug combinations and schedules remain to be defined and there is hitherto no world-wide accepted standard regimen. Cisplatin, an alkylating agent with high putative toxicity is currently widely used although its effectiveness in this disease has not been established firmly. We conducted a meta-analysis of published data reporting trials randomizing a cisplatin-containing regimen versus a regimen without this alkylating agent in order to determine possible differences in survival response and toxicity. Nineteen trials have been identified in medical literature (4054 evaluable patients). Ten trials randomized patients to receive a cisplatin-etoposide regimen versus a regimen without any of these two drugs. A subgroup analysis was, therefore, carried out in the nine remaining trials that randomly allocated patients between two regimens differing in the absence or presence of cisplatin, whereas etoposide was given (or not given) in both arms (1579 evaluable patients). The DerSimonian and Laird method was used to estimate the size effects and the Peto and Yusuf method was used in order to generate the odds ratios (OR) of reduction in risk of death and the increase in probability of being responders to chemotherapy. There was no significant difference between the cisplatin-containing regimen and the regimen without this drug when the risk of toxic-death was taken into account with respective probabilities of 3.1 and 2.7% (NS). Patients randomized in a cisplatin-containing regimen had an increase in probability of being responders with an OR of 1.35, 95% confidence interval (CI) of 1.18–1.55; $P < 10^{-5}$ corresponding to an increase of objective (partial plus complete) response rate from 0.62 to 0.69 (a result taking into account a significant heterogeneity). Patients treated with a cisplatin-containing regimen benefited from a significant reduction of risk of death at 6 months and 1 year with respective OR 0.87, 95% CI 0.75–0.98, $P = 0.03$, and or 0.80, 95% CI 0.69–0.93, $P = 0.002$ (no statistical heterogeneity). This corresponded to a significant increase in the probability of survival of 2.6% and 4.4% at 6 months and 1 year respectively. The meta-analysis restricted to the subset of nine trials without etoposide treatment imbalance reached similar conclusions. A cisplatin-containing regimen yields a higher response rate and probability of survival than does a chemotherapy containing others alkylating agents without a perceptible increase in risk of toxic-death. © 2000 Cancer Research Campaign

**Keywords**: SCLC; chemotherapy; cisplatin; meta-analysis

Chemotherapy is the main treatment for small-cell lung cancer (SCLC), one of the solid tumours which responds best to cytotoxic agents, and survival benefit has been widely demonstrated (Hansen et al, 1991). Various drug combinations have been used in sequential chemotherapies in order to improve both response rate and survival. Among these combinations, cisplatin and etoposide (Ihde et al, 1984; Maksymiuk et al, 1994; Evans et al, 1985) or cyclophosphamide, doxorubicin and vincritine (Feld et al, 1989) are two widely applied regimens inducing a 80–90% overall response rate including a 30–40% complete response rate. Despite the high chemosensitivity of SCLC, the 2- and 5-year survivals are low due to the frequent occurrence of chemoresistant relapses after these classical regimens. In SCLC, relapses are presumed to be have a relationship with the lack of efficiency of the induction treatment which selects sub-clones of the tumour sharing genetic resistance to cytotoxic agents. The search to improve the SCLC chemotherapy outcome aims to circumvent these secondary chemoresistances. Optimal chemotherapy drug combinations and schedules therefore remain to be established.

Cisplatin (*cis*-diamine-dichloroplatinum II [$N_2Cl_2PtH_6$]) is a planar inorganic heavy metal compound yielding alkylating properties (Rosenberg et al, 1965). It does not possess a cell-cycle dependency and is active throughout the cell cycle. This cytotoxic agent is a pivotal drug in the treatment of many human malignancies such as non-seminomatous germinal cell tumour (Einhorn and Donohue, 1977), lymphoma (Rossof et al, 1972) and ovarian cancer (Wiltshaw and Kroner, 1976). In non-small-cell lung cancer (NSCLC), randomized trials have suggested that cisplatin might also be an important drug. For instance, in stage III NSCLC, meta-analysis of randomized studies comparing a chemotherapy–radiotherapy

*Received 28 June 1999*
*Revised 1 February 2000*
*Accepted 17 February 2000*

*Correspondence to:* J-L Pujol

*Present address: Cliniques Universitaires UCL de Mont-Godinne, B 5530 Yvoir, Belgium combination versus radiotherapy alone has demonstrated that the combined modality treatment reduces the risk of death. This effect is particularly apparent where the chemotherapy is based on a modern combination. It is noteworthy that the break between older and more modern regimens coincided with the introduction of cisplatin in NSCLC chemotherapy (Non-Small Cell Lung Cancer Collaborative Group, 1995). Although chemotherapy is supposed to yield a better survival impact in SCLC than in NSCLC, the role of cisplatin in the former histology is still unclear.

In SCLC, cisplatin is widely used although its role in improving patient survival has not been firmly established. As this drug also yields a putatively high toxicity (mainly nephrotoxicity, ototoxicity and emesis), it might be of interest to determine whether or not the use of cisplatin is supported by evidence of effectiveness in SCLC.

A meta-analysis of published randomized trials comparing a cisplatin-containing regimen with a combination without cisplatin was made in an attempt at determining whether or not this alkylating agent improves response and survival.

### TRIALS AND METHODS

### Eligibility criteria

To be included in this meta-analysis, trials fulfilled the following criteria; randomized trials involving previously untreated and histologically or cytologically proven SCLC patients with comprehensible randomization methods (particularly allocation concealment); randomized allocation of patients between a cisplatin-containing regimen and a regimen without this alkylating agent. Trials comparing an alternating approach of two chemotherapies one of them containing cisplatin versus a single regimen without cisplatin were also eligible. Studies comparing a cisplatin-containing regimen to chemotherapy based upon another platinum cytotoxic agents (particularly carboplatin) were not eligible. In addition to the above-mentioned key criteria, a number of quality-control items of publication were taken into account, in particular definition of hypothesis in the statistics section of each article, definition of the patients' characteristics in regard to fundamental prognostic factors (sex, performance index, stage of the disease), definition of the treatment protocol and drug dosage reduction procedures and definition of survival. Trials were also screened regarding the report of the number of evaluable patients, response assessment procedure and toxicity, particularly toxic deaths. Finally, the lack of confusing additional variables such as imbalance of radiotherapy between the two regimens and the number of patients lost to follow-up were checked.

### Selections of trials

A computerized bibliography was extracted from MEDLINE and CANCERLIT (CancerNet™) databases using medical subject headings of the following terms: lung neoplasm, small-cell cancer, randomized trials, chemotherapy, cisplatin or cisplatinum or *cis*-diamine-dichloroplatinum. The search was carried out from 1966 to the end of 1999 inclusive. Afterwards, the manual selection of relevant trials was based upon summary analysis. In addition to the above-mentioned procedure, bibliographies of selected full papers were screened in order to disclose other relevant articles and SCLC experts were consulted about the topic in order to make the meta-analysis as exhaustive as possible. We did not disclose any unpublished trial matching the selection criteria.

### Collection of data

The following general items were recorded: year of publication, hypothesis, method of randomization and method of analyses (intent to treat or fully-eligible population). In addition, the following variables were recorded for each arm of treatment: number of accrual patients, number of eligible patients, number of patients lost to follow-up, mean age, sex ratio, proportion of patients with good performance status (0 or 1), extensive versus limited disease stage. Regarding treatment design we input the precise drugs in each arm, and in the cisplatin-containing regimen, the planned dose-intensity for this drug in mg $m^{-2}$ $week^{-1}$. Finally, the number of patients receiving thoracic radiotherapy was also recorded.

The outcome of the meta-analysis for each treatment arm was assessed as follows: number of toxic-deaths (other toxicities not having been homogeneously reported), percentage of patients achieving a partial or complete response, overall survival at 6 months and 1 year. The latter two parameters were directly graphically measured from magnifications of publication graphs. When the data were directly reported in the text, comparisons with graphic assessment were in good agreement. An attempt to contact the first author of each selected article was made in order to obtain authorization to use the data and to know whether there has been any update of the trial following its publication. Unfortunately, due to the change in position of several colleagues this could not be achieved exhaustively.

### Statistical analysis

Two methods were used in order to estimate the effects of cisplatin on survival, response and toxicity of SCLC patients. The Yusuf and Peto method produces odds ratio (OR) and its 95% confidence interval (CI) together with the value of the heterogeneity test (Yusuf et al, 1985). In addition, the DerSimonian and Laird method was used in order to estimate the size effects upon the different parameters and their 95% CIs were calculated (DerSimonian and Laird, 1986). For this method the value and the 95% CI were corrected taking into account the heterogeneity where this latter parameter was statistically significant.

### RESULTS

A total of 19 trials fulfilled the criteria of selection (Table 1; Eagan et al, 1981; Fukuoka et al, 1986, 1991; Evans et al, 1987; Haveman et al, 1987; Wolf et al, 1987; Chahinian et al, 1989; Sculier et al, 1990*a*, 1993; Goodman et al, 1990; Smith et al, 1990; Wampler et al, 1991; Kanitz et al, 1992; Roth et al, 1992; Farris et al, 1993; Sculier et al, 1993; Joss et al, 1994; Veronesi et al, 1994; Souhami et al, 1997; Urban et al, 1999). One trial was rejected due to the fact that patients had received chemotherapy prior to randomization as it was a second-line chemotherapy study (O'Bryan et al, 1990). A total of 4054 eligible patients were randomized between a cisplatin-containing regimen (1814 patients) and a regimen without cisplatin (2240 patients). Since the beginning of the 1980s it has been hypothesized that the cisplatin–vepeside combination generates a synergistic activity. Some studies selected for the

© 2000 Cancer Research Campaign

**Table 1** Trials of cisplatin-containing regimen versus a regimen without cisplatin

| Publications | Drugs | Planned dose intensity for cisplatin (mg $m^{-2}$ $week^{-1}$) | Number of evaluable patients | Percent of male gender | PS0-1 | Number of limited/ extensive diseases | Number of patients receiving radiotherapy |
|---|---|---|---|---|---|---|---|
| Eagan et al, 1981 | CDDP, VP-16, CTX, DXR, VCR, | 10 | 31 | 0.74 | 27 | 31/0 | 28 |
| | CTX, DXR, VCR, VP-16 | | 31 | 0.70 | 26 | 31/0 | 27 |
| Fukuoka et al, 1986 | CDDP, DXR, VP-16, CTX, VCR, ACNU, PCZ | 15 | 35 | 0.80 | 19 | 10/25 | 6 |
| | CTX, VCR, ACNU; PCZ | | 34 | 0.76 | 23 | 11/23 | 5 |
| Wolf et al, 1987 | CDDP, VP-16 | 27 | 73 | 0.89 | 57 | 27/46 | 23 |
| | IFO, VP-16 | | 68 | 0.91 | 53 | 21/47 | 23 |
| Haveman et al, 1987 | CDDP, DXR, VCR, VP-16, VDS, IFO, CTX, MTX, CCNU | 15 | 150 | 0.84 | 120 | 51/99 | 53 |
| | CTX, DXR, VCR | | 152 | 0.84 | 116 | 53/99 | 32 |
| Evans et al, 1987 | CDDP, VP16, VCR, CTX, DXR | 25 | 145 | 0.65 | 92 | | 114 |
| | CTX, DXR, ADR, VCR | | 144 | 0.68 | 77 | | 108 |
| Chahinian et al, 1989 | CDDP, MTC, VP-16, HXM, MTX; DXR, CTX; CCNU | 5 | 105 | 0.63 | 69 | 0/105 | 17 |
| | MTX, DXR, CTX, CCNU | | 86 | 0.72 | 60 | 0/86 | 10 |
| | MTX, DXR, CTX, CCNU + Warfarin | | 103 | 0.68 | 68 | 0/103 | 2 |
| Sculier et al, 1990 | CDDP, VP16, VDS | 20 | 85 | 0.89 | 57 | 32/53 | 18 |
| | VP16, VDS | | 98 | 0.90 | 68 | 45/53 | 13 |
| Goodman et al, 1990 | CDDP, VP-16, CTX; DXR, VCR | 16.6 | 194 | 0.68 | 163 | 194/0 | – |
| | VP-16, VCR, DXR, CTX | | 194 | 0.58 | 157 | 194/0 | – |
| Smith et al, 1990 | CDDP, VP16 | 33 | 47 | 0.74 | – | 30/17 | 6 |
| | CTX, DXR, VDS, VP-16 | | 48 | 0.56 | – | 30/18 | 6 |
| Fukuoka et al, 1991 | CDDP, VP-16 | 26.6 | 97 | 0.80 | 67 | 47/50 | 35 |
| | CTX, DRX, VCR | | 97 | 0.83 | 62 | 49/48 | 41 |
| | CDDP, VP-16, CTX, DXR, VCR | | 94 | 0.81 | 66 | 50/44 | 43 |
| Wampler et al, 1991 | MTX, CDDP, VP-16, CTX, DXR, VCR | 13.6 | 82 | 0.71 | 63 | 0/82 | – |
| | CTX, DXR, VCR | | 82 | 0.67 | 63 | 0/82 | – |
| Kanitz et al, 1992 | CDDP, epi-DXR | 15 | 59 | 0.88 | 25 | 0/59 | – |
| | EpiDXR, CTX | | 52 | 0.81 | 34 | 0/52 | – |
| Roth et al, 1992 | CDDP, VP-16 | 33 | 148 | 0.77 | 84 | 0/148 | – |
| | CTX, DXR, VCR | | 146 | 0.80 | 84 | 0/146 | – |
| | CDDP, VP-16, CTX, DXR, VCR | | 143 | 0.75 | 79 | 0/143 | – |
| Sculier et al, 1993 | CDDP, VP-16, CTX, DXR, VCR, VDS, MTX | 20 | 98 | 0.90 | 79 | 47/60 | 31 |
| | VP-16, CTX, DXR | | 101 | 0.90 | 85 | 41/60 | 21 |
| Farris et al, 1993 | CDDP, VP-16, CTX, epi-DXR | 25 | 45 | 0.89 | 56 | 14/31 | 14 |
| | CTX, epi-DXR, VCR | | 57 | 0.88 | 57 | 27/30 | 9 |
| Veronesi et al, 1994 | CDDP, VP-16 | 33 | 70 | 0.86 | – | 22/48 | 42 |
| | CTX, epi-DXR, VCR | | 66 | 0.89 | – | 33/33 | 42 |
| Joss et al, 1994 | CDDP, VP-16, DXR | 15 | 92 | 0.87 | 69 | 41/51 | |
| | CTX, VP-16, DXR | | 86 | 0.89 | 64 | 38/48 | 49 |
| | CDDP, VP-16, DXR, MTX, CTX, VCR | | 88 | 0.91 | 68 | 38/50 | |
| Souhami et al, 1997 | CDDP, VP-16, CTX, DXR, VCR | 20 | 67 | 0.52 | 35 | 7/60 | – |
| | VP-16 | | 67 | 0.55 | 39 | 8/59 | – |
| Urban et al, 1999 | CDDP, VP-16, CTX, DXR, CCNU, VDS | 10 | 191 | 0.91 | 99 | 81/110 | – |
| | CTX, DXR, CCNU | | 203 | 0.93 | 103 | 88/115 | – |

ACNU: nimustine hydrochloride; CCNU: carmustine; CDDP: cisplatin; CTX: cyclophosphamide; HXM: hexamethylmelamine; DXR: doxorubicin; epi-DXR: epidoxorubicin; IFO: ifosfamide; MTC: mitomycin C: MTX: methotrexate; PCZ: procarbazine; VCR: vincristine; VDS: vindesine; VP-16: vepeside.

*© 2000 Cancer Research Campaign*

**Table 2** Results of DerSimonian and Laird meta-analysis of trials of cisplatin containing regimen versus a regimen without cisplatin

| | Cisplatin-containing regimen (Probability) | Regimen without cisplatin *(Probability)* | Y[b] | Y: 95% confidence interval | *P* | Heterogeneity | Degrees of freedom | p heterogeneity |
|---|---|---|---|---|---|---|---|---|
| Toxic death rate | 0.031 | 0.027 | 0.004 | –0.007–0.016 | 0.23 | Q=15.37 | 15 | 0.41 |
| Response rate | 0.688 | 0.619 | 0.070[a] | 0.021–0.125[a] | <0.0001 | Q=51.08 | 18 | 0.001 |
| Survival at 6 months | 0.684 | 0.658 | 0.026 | 0.001–0.055 | 0.030 | Q=22.28 | 18 | 0.22 |
| Survival at 1 year | 0.288 | 0.244 | 0.044 | 0.0016–0.072 | 0.0009 | Q=25.85 | 18 | 0.15 |

[a] Corrected value taking into account a significant heterogeneity. [b] Size effect according to the DerSimonian and Laird method.

meta-analysis actually randomized patients between a cisplatin–vepeside combination (sometimes included in an alternating chemotherapy programme) versus a regimen with neither of these two drugs. Thus for these trials there was a vepeside administration imbalance between the two regimens. Therefore, a subset meta-analysis was made for the nine studies without vepeside imbalance (i.e. vepeside administered similarly in both arms or not given in either arm). These trials included 1579 evaluable patients (Eagan et al, 1981; Wolf et al, 1987; Goodman et al, 1990; Sculier et al, 1990*a* 1993; Smith et al, 1990, Kanitz et al, 1992; Joss et al, 1994; Souhami et al, 1997).

### Toxicity

Data regarding neutropenic infections, nephrotoxicity and ototoxicity were not reported homogeneously among the different trials. On the other hand, the number of toxic-deaths was clearly reported by 16 out of the 19 trials. Therefore, we focused on the percentage of patients affected by a toxic-death. Patients treated with a cisplatin-containing regimen were not at higher risk of toxic death than patients treated with a regimen without this alkylating agent with respective probabilities of 0.031 and 0.027 and a size effect of 0.004 according to the DerSimonian and Laird method (Table 2).

### Response

Patients who had received a cisplatin-containing regimen proved to have a higher probability of being responders in comparison with patients included in a regimen without cisplatin, with an OR (95% confidence interval (CI)) of 1.35 (95% CI 1.18–1.55); $P < 10^{-5}$. This result corresponded to an OR of being non-responder of 0.74 (95% CI 0.85–0.64) for patients receiving cisplatin-containing regimens (Figure 1). With these regimens, the DerSimonian and Laird method measured a significant ($P < 0.0001$) increase in response rate from 0.62 to 0.69 taking into account a significant heterogeneity (Q = 51.08; degree of freedom: 18: $P < 0.05$).

### Survival

Patients treated with a cisplatin-containing regimen proved to have a lower risk of death in comparison with patients treated by a regimen without cisplatin at 6 months and 1 year with a respective OR of 0.87 (95% CI 0.75–0.98); $P = 0.03$ (Figure 2) and OR 0.80 (95% CI 0.69–0.93); $P = 0.002$ (Figure 3) without statistical heterogeneity. The corresponding survival rates at 6 months were 0.68 and 0.65 for the former and latter type of chemotherapy as measured by the DerSimonian and Laird method. These rates were 0.29 and 0.24 at 1 year l 2). This corresponded to a significant increase in the probability of survival of 2.6% and 4.4% at 6 months and 1 year respectively.

### Subgroup analysis in trials without vepeside imbalance

Nine trials without vepeside administration imbalance between the cisplatin-containing regimen and the regimen without cisplatin were meta-analysed and the size effects measured using the DerSimonian and Laird method (Table 3). All conclusions drawn from the meta-analysis of the whole selection of trials were also applicable to this subset analysis. In particular, patients receiving a cisplatin-containing regimen proved to have a lower risk of death at 6 months with an OR of 0.74 (95% CI 0.59–0.94); $P = 0.006$. At 1 year the OR was 0.80 (95% CI 0.63–0.98); $P = 0.03$. The DerSimonian and Laird method showed a significant 4.4% survival benefit at 6 months and 4.1% at 1 year in favour of the cisplatin-containing regimen (Table 3). The OR of being a responder in a cisplatin-containing regimen was 1.48 (95% CI 1.18–1.87); $P = 0.01$.

### DISCUSSION

In this meta-analysis, the putative benefits of a cisplatin regimen were analysed in a selection of trials comparing a cisplatin-containing regimen and chemotherapy based on other alkylating agents. Patients receiving a cisplatin regimen proved to have a better probability of being responders, and a better survival. These effects were of moderate size. However, no detectable increase in the risk of toxic death was disclosed.

It is widely accepted that prognosis of SCLC is improved by chemotherapy (Hansen et al, 1991). Combination chemotherapy was the main treatment in the 1970s. Multiple drug combinations led to a longer survival than single drug treatment or radiation-therapy alone. Among the active drugs in SCLC, cyclophosphamide–adriamycin–vincristine (Fukuoka et al, 1986; Feld et al, 1989) or cyclophosphamide–adriamycin–etoposide combinations have long been known as having a good efficacy/toxicity ratio. Other combination chemotherapies such as cisplatin and etoposide have been tested successfully and have given encouraging results in terms of both response and survival rates (Ihde et al, 1984; Maksymiuk et al, 1994; Evans et al, 1985). It rapidly appeared that response to chemotherapy is one of the most important factors for SCLC patients to achieve long-term survival (Osterlind et al, 1986) in association with other known favourable prognostic factors, namely limited disease, good performance status, lower age group, low serum lactate dehydrogenase, low alkaline phosphatases and normal serum sodium and bicarbonate (Cerny et al, 1987). Other investigators have demonstrated

© 2000 Cancer Research Campaign 

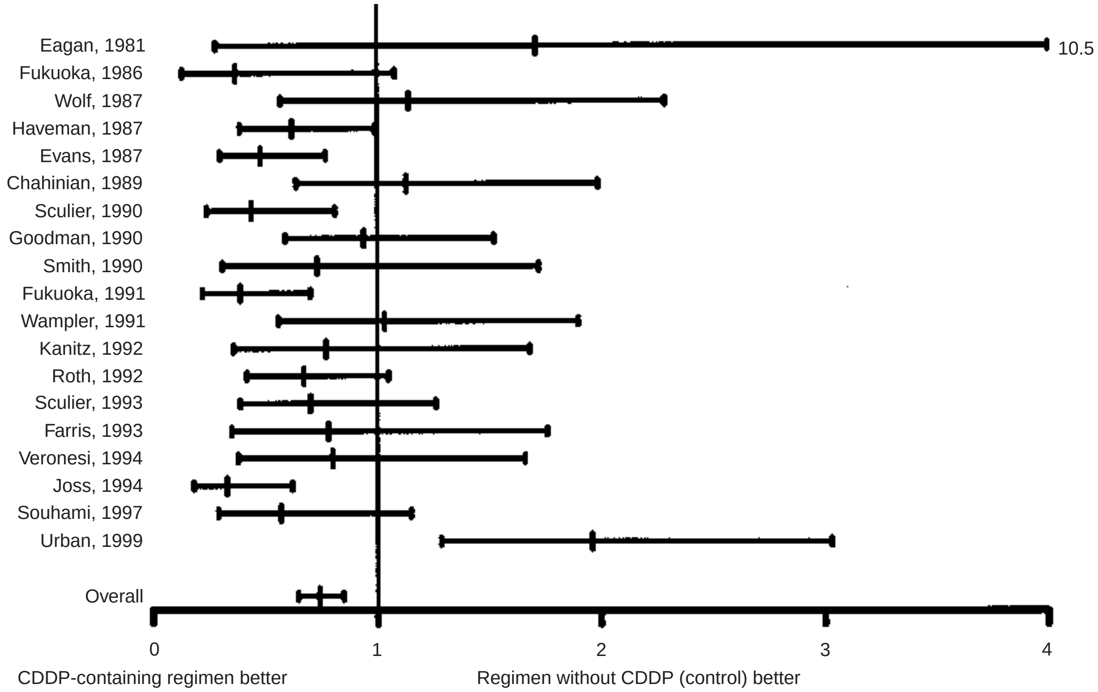

**Figure 1** Odds ratio and 95% confidence interval of being a non-responder for patients treated with a CDDP-containing regimen ($P < 10^{-5}$). Results are expressed as individual and overall ORs, vertical bar, and their respective 95% confidence intervals, horizontal bar. ORs lower than 1 indicate a reduction of risk of being non-responders with a cisplatin-containing regimen (overall OR (95%CI): 0.74 (0.85–0.64). Test for heterogeneity: Q = 46.01; df: 18; $P = 0.001$

that the cisplatin–etoposide combination is efficient as a rescue treatment for patients who relapsed after cyclophosphamide–adriamycin–vincristine, by inducing a 50% response rate in these poor conditions of second-line treatment (Evans et al, 1985; Porter et al, 1985). Conversely, patients previously treated with cisplatin–etoposide might respond to cyclophosphamide–adriamycin–vincristine combination, although in a lower proportion (10–20%) (Sculier et al, 1990*b*). This indirect comparison suggests a superiority of the cisplatin-based regimen. The meta-analysis presented here adds another clue in favour of the early use of cisplatin in the course of SCLC.

One may hypothesize that, due to the nature of the herein meta-analysis based on published data, a possible bias was introduced insofar as our procedure did not allow the disclosure of unpublished trials. A direct comparison of meta-analysis on medical literature and meta-analysis on individual patient data has been performed in the setting of cisplatin-based chemotherapy in ovarian cancer (Stewart and Parmar, 1993). This study suggested possible differences in estimated treatment effect due to patient exclusions and shorter length of follow-up in the former technique. On the other hand, regarding survival, which was the main outcome of our meta-analysis, there was no statistical heterogeneity. In addition, there was no apparent effect of epoch when year of publication was taken into account. In particular, considering the 1-year survival outcome, four trials did not detect any difference between the two types of treatment, three others were able to demonstrate the statistical benefit conferred by a cisplatin-based regimen, whereas the remaining 12 trials suggested a trend (nine in favour of cisplatin containing regimens and three in favour of chemotherapy without this alkylating agent). The results of the meta-analysis are therefore in agreement with a qualitative analysis of the literature which was in favour of the beneficial effect of cisplatin.

Vepeside–cisplatin has been widely used since the 1980s as a control regimen owing to the putative synergy of this combination. We therefore investigated whether or not the cisplatin benefit such as it was observed in the whole selection of trials was linked to the synergistic action of the cisplatin–vepeside combination. As shown by the subset analysis made in the subgroup of nine trials without vepeside imbalance, cisplatin-containing regimens shared an advantageous effect on both survival and response. Thus, the administration of cisplatin seems to result in an improvement of outcome as a result of its administration per se.

Finally, our meta-analysis did not include any trial involving a carboplatin-containing regimen. This choice was made for two reasons: first, carboplatin yields a different pattern of toxicity, resulting in a putative difference in combination effect; secondly, randomized trials involving this drug administered in one arm only, mainly compared carboplatin with cisplatin. Thus, our conclusions apply to cisplatin only and cannot be extended to other platinum agents.

Although modest, the survival improvement observed in cisplatin-containing chemotherapy as measured in comparison with chemotherapy based upon other alkylating agents allows the recommendation of the former regimen as a control arm in future chemotherapy research. Important questions remain unanswered in particular that of the optimal dosage of cisplatin and the choice of drugs it should be correctly associated with.

### ACKNOWLEDGEMENTS

The authors wish to thank Mrs Jo Baïssus for help in preparing the manuscript. Supported by a grant from the French League Against Cancer (Hérault committee).

© 2000 Cancer Research Campaign

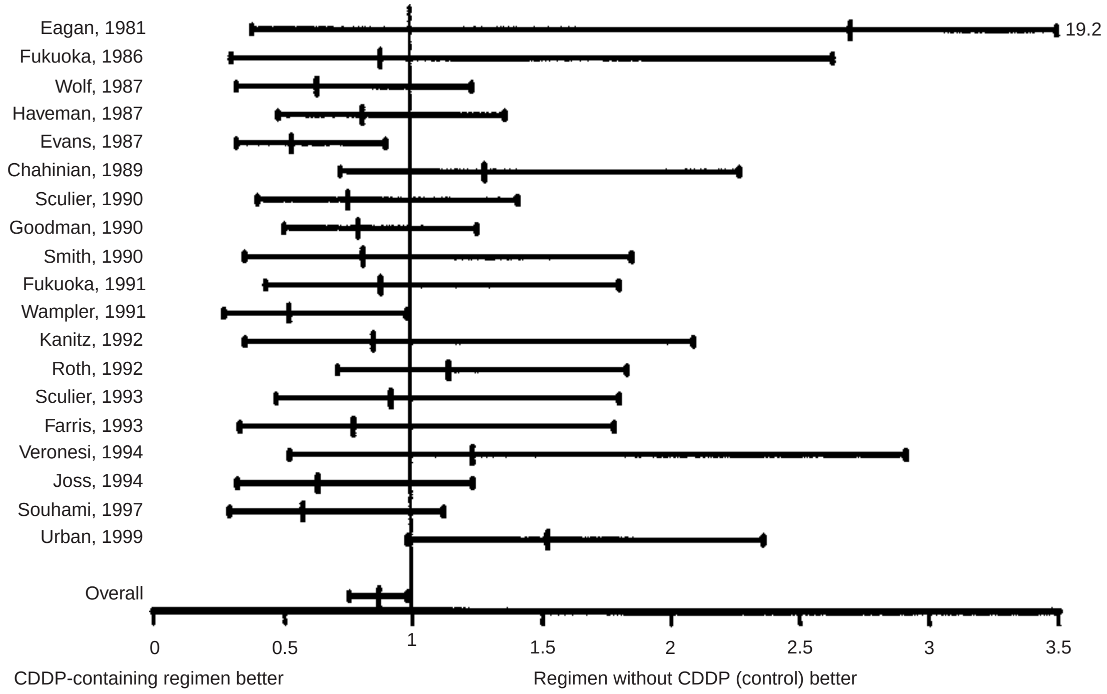

**Figure 2** Odds ratio and 95% confidence interval of mortality at 6 months for patients treated with a CDDP-containing regimen (symbols as in Figure 1; $P = 0.03$). Test for heterogeneity: Q = 21.68; df: 18; $P = 0.25$

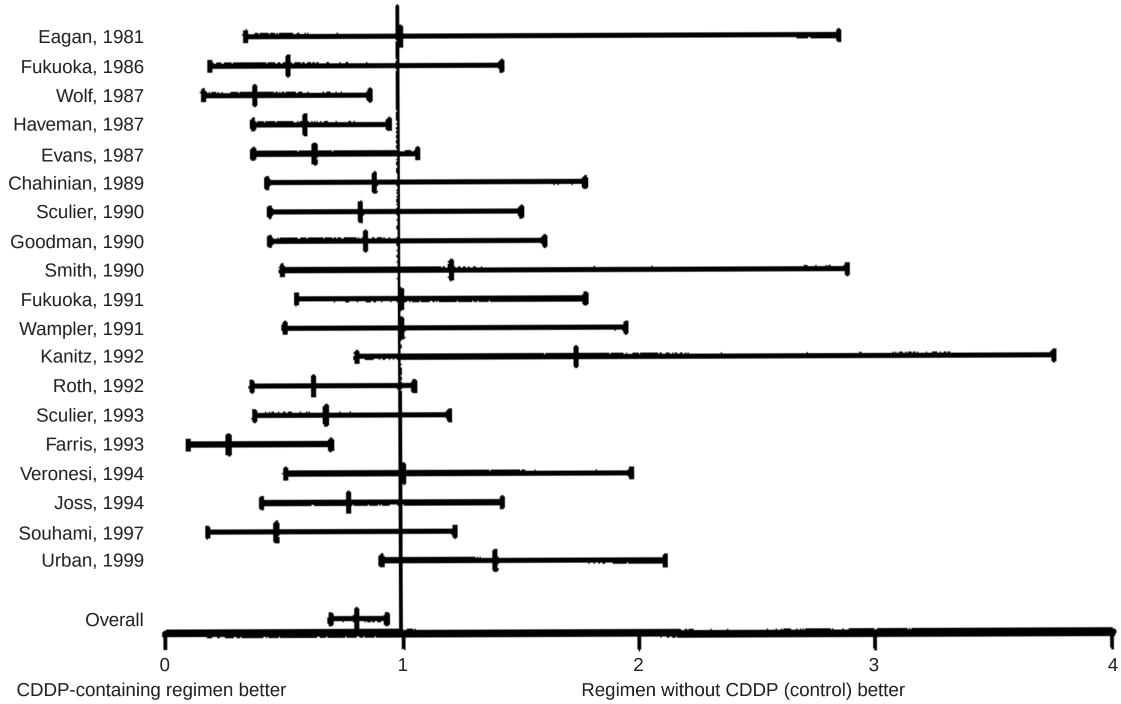

**Figure 3** Odds ratio and 95% confidence interval of mortality at 1 year for patients treated with a CDDP-containing regimen (symbols as in Figure 1; $P = 0.002$). Test for heterogeneity: Q = 26.47; df: 18; $P = 0.10$

*© 2000 Cancer Research Campaign* 

**Table 3** Results of Laird and DerSimonian meta-analysis of trials of cisplatin-containing regimen versus a regimen without cisplatin (restricted to nine trials with etoposide balance)

| | Cisplatin-containing regimen (Probability) | Regimen without cisplatin (Probability) | Y[b] | Y: 95% confidence interval | *P* | Heterogeneity | Degrees of freedom | p heterogeneity |
|---|---|---|---|---|---|---|---|---|
| Toxic death rate | 0.0249 | 0.0239 | 0.001 | –0.016–0.017 | 0.45 | Q=8.91 | 7 | 0.25 |
| Response rate | 0.737 | 0.667 | 0.077[a] | 0.0116–0.143[a] | 0.001 | Q=16.05 | 8 | 0.03 |
| Survival at 6 months | 0.628 | 0.583 | 0.044 | 0.002–0.087 | 0.02 | Q=5.73 | 8 | 0.75 |
| Survival at 1 year | 0.244 | 0.203 | 0.041 | 0.0006–0.080 | 0.02 | Q=9.61 | 8 | 0.28 |

[a] Corrected value taking into account a significant heterogeneity. [b] Size effect according to the DerSimonian and Laird's method.

### REFERENCES

Cerny T, Blair V, Anderson H, Bramwell V and Thatcher N (1987) Pretreatment prognostic factors and scoring system in 407 small-cell lung cancer patients. *Int J Cancer* **15**: 146–149

Chahinian P, Propert K, Ware J, Zimmer B, Perry M, Hirsch V, Skarin A, Kopel S, Holland J, Comis R and Green M (1989) A randomized trial of anticoagulation with warfarin and of alternating chemotherapy in extensive small-cell lung cancer by the Cancer and Leukemia Group B. *J Clin Oncol* **8**: 993–1002

DerSimonian R and Laird N (1986) Meta-analysis in clinical trials. *Control Clin Trials* **7**: 177–188

Eagan R, Lee R, Frytak S, Fleming T, Ingle JN, Creagan ET, Nichols WC, Kvols LK and Coles DT (1981) An evaluation of low-dose cisplatin as part of combined modality therapy of limited small cell lung cancer. *Cancer Clin Trials* **4**: 267–271

Einhorn LH and Donohue J (1977) Cis-diamminedichloroplatinum, vinblastine and bleomycin. Combination chemotherapy in disseminated testicular cancer. *Ann Inter Med* **87**: 293–298

Evans WK, Shepherd FA, Feld D, Osoba D, Dang P and DeBoer G (1985*a*) VP-16 and cisplatin as first line therapy for small-cell lung cancer. *J Clin Oncol* **3**: 1471–1477

Evans, WK, Osoba, D, Feld, R, Shepherd, FA, Bazos MJ and De Boer, G (1985*b*) Etoposide (VP 16) and cisplatin: an effective treatment for relapse in small-cell lung cancer. *J Clin Oncol* **3**: 65–71

Evans W, Feld R, Murray N, Willan A, Coy P, Osoba D, Shepherd F, Clark D, Levitt M, MacDonald A, Wilson K, Shelley W and Pater J (1987) Superiority of alternating non-cross-resistant chemotherapy in extensive small-cell lung cancer. A Multicenter, Randomized Clinical Trial by the National Cancer Institute of Canada. *Ann Intern Med* **107**: 451–458

Farris A, Bisail M, Sarobba MG, Sanna G, Scotto T, Valzelli S and Intini C (1993) Cisplatin-VP16 alternating with cyclophosphamide–epirubicin versus cyclophosphamide– epirubicin–vincristine in small cell lung cancer. *J Chemother* **5**: 344–347

Feld R, Evans WK, De Boer G, Quirt IC, Shepherd FA, Yeoh JL, Pringle JF, Payne DG, Herman JG and Chamberlain D (1984) Combined modality induction therapy without maintenance chemotherapy for small-cell carcinoma of the lung. *J Clin Oncol* **2**: 294–304

Fukuoka M, Takada M, Negoro S, Kusunoki Y, Matsui K, Ryu S, Sakai N, Takifuji N, Kudoh S and Tamai S (1986) Alternating non-cross resistant chemotherapy for small-cell lung cancer. *Jpn J Clin Oncol* **16**: 261–270

Fukuoka M, Furuse K, Saijo N, Nishiwaki Y, Ikegami H, Tamura T, Shimoyama M and Suemasu K (1991) Randomized trial of cyclophosphamide, doxorubicin, and vincristine versus cisplatin and etoposide versus alternation of these regimens in small-cell lung cancer. *J Natl Cancer Inst* **83**: 855–861

Goodman G, Crowley J, Blasko J, Livingston R, Beck T, Demattia M and Bukowski R (1990) Treatment of limited small-cell lung cancer with etoposide and cisplatin alternating with vincristine, doxorubicin, and cyclophosphamide versus concurrent etoposide, vincristine, doxorubicin, and cyclophosphamide and chest radiotherapy: a Southwest Oncology Group Study. *J Clin Oncol* **8**: 39–47

Hansen HH and Kristjansen PEG (1991) Chemotherapy of small-cell lung cancer. *Eur J Cancer* **27**: 342–349

Havemann C, Wolf M, Holle R, Gropp C, Drings P, Manke H, Hans K, Schroeder M, Heim M, Victor N, Georgii A, Thomas C, Pflüger K and Bepler G (1987) Alternating versus sequential chemotherapy in small cell lung cancer. A randomised German Multicenter Trial. *Cancer* **59**: 1072–1082

Ihde DC, Mulshine JL, Kramer BS, Steinberg SM, Linnoila RI, Gazdar AF, Edison M, Phelps RM, Lesar M and Phares JC (1984) Prospective randomized comparison of high-dose and standard-dose etoposide and cisplatin chemotherapy in patients with extensive-stage small cell lung cancer. *J Clin Oncol* **12**: 2022–2034

Joss R, Alberto P, Bleher E, Ludwig C, Siegenthaler P, Martinelli G, Sauter C, Schatzmann E and Senn H (1994) Combined-modality treatment of small-cell lung cancer: randomized comparison of three induction chemotherapies followed by maintenance chemotherapy with or without radiotherapy of the chest. *Ann Oncol* **5**: 921–928

Kanitz E, Kolaric K, Jassem J, Mechl Z, Pawlicki M, Ringwald G, Rolski J, Schoket Zs, Vukas D, Kaplan E and Eckhardt S (1992) Randomized phase II trial of high-dose 4´-epi-doxorubicin plus cyclophosphamide versus high-dose 4´-epi-doxorubicin plus cisplatin in previously untreated patients with extensive small cell lung cancer. *Oncology* **49**: 327–332

Maksymiuk AW, Jett JR, Earle JD, Su JQ, Diegert FA, Mailliard JA, Kardinal CG, Krook JE, Veeder MH and Wiesenfeld M (1994) Sequencing and schedule effects of cisplatin plus etoposide in small cell lung cancer: results of a North Central Cancer Treatment Group randomized clinical trial. *J Clin Oncol* **12**: 70–76

Non-Small Cell Lung Cancer Collaborative Group (1995) Chemotherapy in non-small-cell lung cancer: a meta-analysis using updated data on individual patients from 52 randomized clinical trials. *Br Med J* **311**: 899–909

O'Bryan RM, Crowley JJ, Kim PN, Epstein RB, Neilan B, Coltman CA, W Stuckey WJ and Pazdur R (1990) Comparison of etoposide and cisplatin with bis-chloroethylnitrosourea, thiotepa, vincristine, and cyclophosphamide for salvage treatment in small cell lung cancer. *Cancer* **65**: 856–860

Osterlind, K, Hansen, HH, Hansen, M, Dombernowsky, P and Andersen, PK (1986) Long-term disease-free survival in small-cell carcinoma of the lung: a study of clinical determinants. *J Clin Oncol* **4**: 1307–1313

Porter, LL III, Johnson, DH, Hainsworth, JD, Hande, KR and Greco, A (1985) Cisplatine and etoposide combination chemotherapy for refractory small-cell carcinoma of the lung. *Cancer Treat Rep* **69**: 479–481

Rosenberg B, Van Camp L and Krigas T (1965) Inibition of cell division in *Eschericia coli* by electrolysis products from a platinum electrode. *Nature* 1965; **205**: 698–699

Rossoff AH, Slayton RE and Perlia CP (1976) Preliminary clinical experience with cisdiamminedichloroplatium (II): a new anti-cancer drug. *Ann Intern Med* **86**: 803–812

Roth B, Johnson D, Einhorm L, Schacter L, Cherng N, Cohen H, Crawford J, Randolph J, Goodlow J, Broun G, Omoura G and Greco A (1992) Randomized study of cyclophosphamide, doxorubicin, and vincristine versus etoposide and cisplatin versus alternation of these two regimens in extensive small-cell lung cancer: a phase III trial of the Southeastern Cancer Study Group. *J Clin Oncol* **10**: 282–291

Sculier JP, Klastersky J, Libert P, Ravez P, Thiriaux J, Lecomte J, Bureau G, Vandermoten G, Dabouis G, Michel J, Schmerber J, Sergysels R, Becquart D, Mommen P and Paesmans M (1990*a*) A randomised study comparing etoposide and vindesine with or without cisplatin as induction therapy for small-cell lung cancer. *Ann Oncol* **1**: 128–133

Sculier, JP, Klastersky J, Libert P, Ravez P, Brohee D, Vandermoten G, Michel J; Thiriaux J; Bureau G and Schmerber J (1990*b*) A phase II study evaluating CAVi (cyclophosphamide, adriamycin, vincristine) potentiated or not by amphotericin B entrapped into sonicated liposomes, as salvage therapy for small-cell lung cancer. *Lung Cancer* **6**: 110–118

Sculier JP, Paesmans M, Bureau G, Dabaouis G, Libert P, Vandermoten G, Van Cutsem O, Berchier MC, Ries F, Michel J, Sergysels R, Mommen P and

 © 2000 Cancer Research Campaign

Klastersky J (1993) Multiple-drug weekly chemotherapy versus standard combination regimen in small-cell lung cancer: a phase III randomized study conducted by the European lung cancer working party. *J Clin Oncol* **11**: 1858–1865

Smith A, Anderson G, Chappell G and Bowen R (1991) Does the substitution of cisplatin in a standard four drug regimen improve survival in small cell carcinoma of the lung? A comparison of two chemotherapy regimens. *Thorax* **46**: 172–174

Souhami RL, Spiro SG, Rudd RM, Ruiz de Elvira MC, James LE, Gower NH, Lamont A and Harper PG (1997) Five-day oral etoposide treatment for advanced small-cell lung cancer: randomized comparison with intravenous chemotherapy. *J Natl Cancer Inst* **89**: 577–580

Stewart LA and Parmar MK (1993) Meta-analysis of the literature or of individual patient data: is there a difference? *Lancet* **341**: 418–422

Urban T, Baleyte T, Chastang CL, Jeannin L, Delaval P, Zaegel M, Mornet M, Coetmeur D and Lebeau B (1999) Standard combination versus alternating chemotherapy in small cell lung cancer: a randomised clinical trial including 394 patients. 'Petites Cellules' Group. *Lung Cancer* **25**: 105–113

Veronesi A, Cartei G, Crivellari D, Magri MD, Della Valentina M, Foladore S, Trovò MG, Nascimben O, Sibau A, Talamini R and Monfardini S (1994) Cisplatin and etoposide versus cyclophosphamide, epirubicin and vincristine in small cell lung cancer: a randomized study. *Eur J Cancer* **30A**: 1474–1478

Wampler G, Heim W, Ellison N, Ahlgren J and Fryer J (1991) Comparison of cyclophosphamide, doxorubicin and vincristine with an alternating regimen of methotrexate, etoposide, and cisplatin/cyclophosphamide, doxorubicin, and vincristine in the treatment of extensive-disease small-cell lung carcinoma: a Mid-Atlantic Oncology Program Study. *J Clin Oncol* **9**: 1438–1445

Wilshaw E and Kroner T (1976) Phase II study of cis-dichlorodiammineplatinum (NSC 119875) in advanced adenocarcinoma of the ovary. *Cancer Treat Rep* **60**: 55–60

Wolf M, Havemann K, Holle R, Gropp C, Drings P, Hans K, Schroeder M, Heim M, Dommes M, Mende S, Thiel H, Hruska D, Victor N, Georgii A and Braun C (1987) Cisplatin/etoposide versus ifosfamide/etoposide combination chemotherapy in small-cell lung cancer: A Multicenter German randomized Trial. *J Clin Oncol* **5**: 1880–1889

Yusuf S, Cellins R, Peto R, Furberg C, Stampfer MJ, Goldhaber SZ and Hennekens CH (1985) Intraveinous and intracoranary fibrinolytic therapy in acute myocardial infarction: overview of results in mortality, reinfarction and side effects for 33 randomised clinical trials. *Eur Heart J* **6**: 556–585

*© 2000 Cancer Research Campaign*
